# Safer Care for Older Persons in (residential) Environments (SCOPE): a pragmatic controlled trial of a care aide-led quality improvement intervention

**DOI:** 10.1186/s13012-022-01259-8

**Published:** 2023-03-29

**Authors:** Adrian Wagg, Matthias Hoben, Liane Ginsburg, Malcolm Doupe, Whitney Berta, Yuting Song, Peter Norton, Jennifer Knopp-Sihota, Carole Estabrooks

**Affiliations:** 1 Division of Geriatric Medicine, Department of Medicine, University of Alberta, Edmonton, Alberta Canada; 2 Faculty of Nursing, University of Alberta, Edmonton, Alberta Canada; 3 School of Health Policy & Management, Faculty of Health, York University, Toronto, Ontario Canada; 4 Departments of Community Health Sciences, Emergency Medicine, Max Rady College of Medicine, Rady Faculty of Health Sciences, University of Manitoba, Winnipeg, Manitoba Canada; 5 Institute of Health Policy, Management & Evaluation, Dalla Lana School of Public Health, University of Toronto, ON Toronto, Canada; 6 Cumming School of Medicine, University of Calgary, Calgary, Alberta Canada; 7 Faculty of Health Disciplines, Athabasca University & Faculty of Nursing, University of Alberta, Edmonton, Alberta Canada

**Keywords:** Long-term care, Care aide, Quality improvement

## Abstract

**Background:**

The increased complexity of residents and increased needs for care in long-term care (LTC) have not been met with increased staffing. There remains a need to improve the quality of care for residents. Care aides, providers of the bulk of direct care, are well placed to contribute to quality improvement efforts but are often excluded from so doing. This study examined the effect of a facilitation intervention enabling care aides to lead quality improvement efforts and improve the use of evidence-informed best practices. The eventual goal was to improve both the quality of care for older residents in LTC homes and the engagement and empowerment of care aides in leading quality improvement efforts.

**Methods:**

Intervention teams participated in a year-long facilitative intervention which supported care aide-led teams to test changes in care provision to residents using a combination of networking and QI education meetings, and quality advisor and senior leader support.

This was a controlled trial with random selection of intervention clinical care units matched 1:1 post hoc with control units. The primary outcome, between group change in conceptual research use (CRU), was supplemented by secondary staff- and resident-level outcome measures. A power calculation based upon pilot data effect sizes resulted in a sample size of 25 intervention sites.

**Results:**

The final sample included 32 intervention care units matched to 32 units in the control group. In an adjusted model, there was no statistically significant difference between intervention and control units for CRU or in secondary staff outcomes. Compared to baseline, resident-adjusted pain scores were statistically significantly reduced (less pain) in the intervention group (*p*=0.02). The level of resident dependency significantly decreased statistically for residents whose teams addressed mobility (*p*<0.0001) compared to baseline.

**Conclusions:**

The Safer Care for Older Persons in (residential) Environments (SCOPE) intervention resulted in a smaller change in its primary outcome than initially expected resulting in a study underpowered to detect a difference. These findings should inform sample size calculations of future studies of this nature if using similar outcome measures. This study highlights the problem with measures drawn from current LTC databases to capture change in this population. Importantly, findings from the trial’s concurrent process evaluation provide important insights into interpretation of main trial data, highlight the need for such evaluations of complex trials, and suggest the need to consider more broadly what constitutes “success” in complex interventions.

**Trial registration:**

ClinicalTrials.gov, NCT03426072, registered August 02, 2018, first participant site April, 05, 2018.

Contributions to the literature

- Given the complexity of care environments and the influence of contextual factors in implementation, pragmatic trials are needed; however, attention to study design and measurement is crucial as is assessment of implementation of complex interventions in the long-term care environment.
- This study, highlights the ways in which a negative trial may still be “successful” despite the absence of anticipated change in the primary outcome, underscoring the need to further explore what constitutes success in complex trials.
- Engagement and empowerment of care aides in leading quality improvement teams can result in tangible improvements in care provided to older adults in LTC.

## Introduction

As a greater proportion of our population survives into late life, the number of people living with chronic and co-existing medical conditions and cognitive impairment has increased [1–5]. Despite policy designed to allow people to age in place, the need for long-term care (LTC) has grown. Annually, 1.7 million older adults in North America reside in LTC homes [6]. Over half of this medically complex, vulnerable population has an accompanying age-related dementia [7–9].

Dementia is one of the most distressing and burdensome health problems encountered by the LTC home workforce [10–14]. Caregivers often associate dementia with increased job strain, reduced job satisfaction, and increased staff turnover [11, 15–19]. This is an ongoing source of concern for families [20–22] and has also resulted in LTC home staff reporting increased workloads and decreased quality of working life [16, 23].

Over recent decades, national and international reports have highlighted concerns about the quality of LTC provided to residents [24–28]. These concerns have been heightened during the COVID-19 pandemic, where residents were disproportionately affected both by the illness itself and by the restrictions placed upon their freedoms, resulting in significant adverse effects [29, 30]. Despite the increasingly complex needs of this highly vulnerable population, increasing proportions of residents with high physical dependency, cognitive impairment, and increasing numbers of co-existing medical conditions [31], LTC home staff levels and skills have not significantly changed over recent years [32, 33]. There is also a long-standing staffing shortfall and difficulty with staff retention which has been exacerbated during the COVID-19 pandemic [34–36].

The vast majority of direct care to residents in LTC homes, including personal care such as bathing, dressing, assistance with mobility and activities of daily living, and increasingly managing bladder and bowel incontinence, wound care and assessment of vital signs, is provided by care aides (also known as personal support workers, nursing aides, nursing assistants) [37, 38]. These unregulated workers seldom have standardized training and have a widely diverse racial composition, often with English as a second language. Among front-line workers, care aides are critically placed to observe, interpret, and respond to residents’ daily needs. They are integral to the provision of quality care [39–41]. Despite calls to include them in care planning and quality improvement initiatives, however, they are routinely excluded, contributing to their beliefs that they are under-valued by others on the team [42–44]. Evidence demonstrates that empowering care aides enhances their work performance and quality of work life [45–47]. However, it is unclear how best to accomplish this and robust intervention studies using systematic, evidence-informed approaches are lacking.

This study examined the effectiveness of the SCOPE (Safer Care for Older Persons in (residential) Environments) intervention. SCOPE is a facilitation and quality improvement intervention which aimed to empower and enable care aides to lead quality improvement (QI) efforts and to improve the quality of care for older residents in LTC homes. We examined whether SCOPE resulted in increased use of best practices in care and improved care aide’s quality of work life. The intervention stemmed from a proof of principle study with care aide led teams [48] which was subsequently subjected to refinement, with care aides, prior to pilot testing [49] in the registered trial reported here (NCT03426072).

### Intervention

**Fig. 1:**
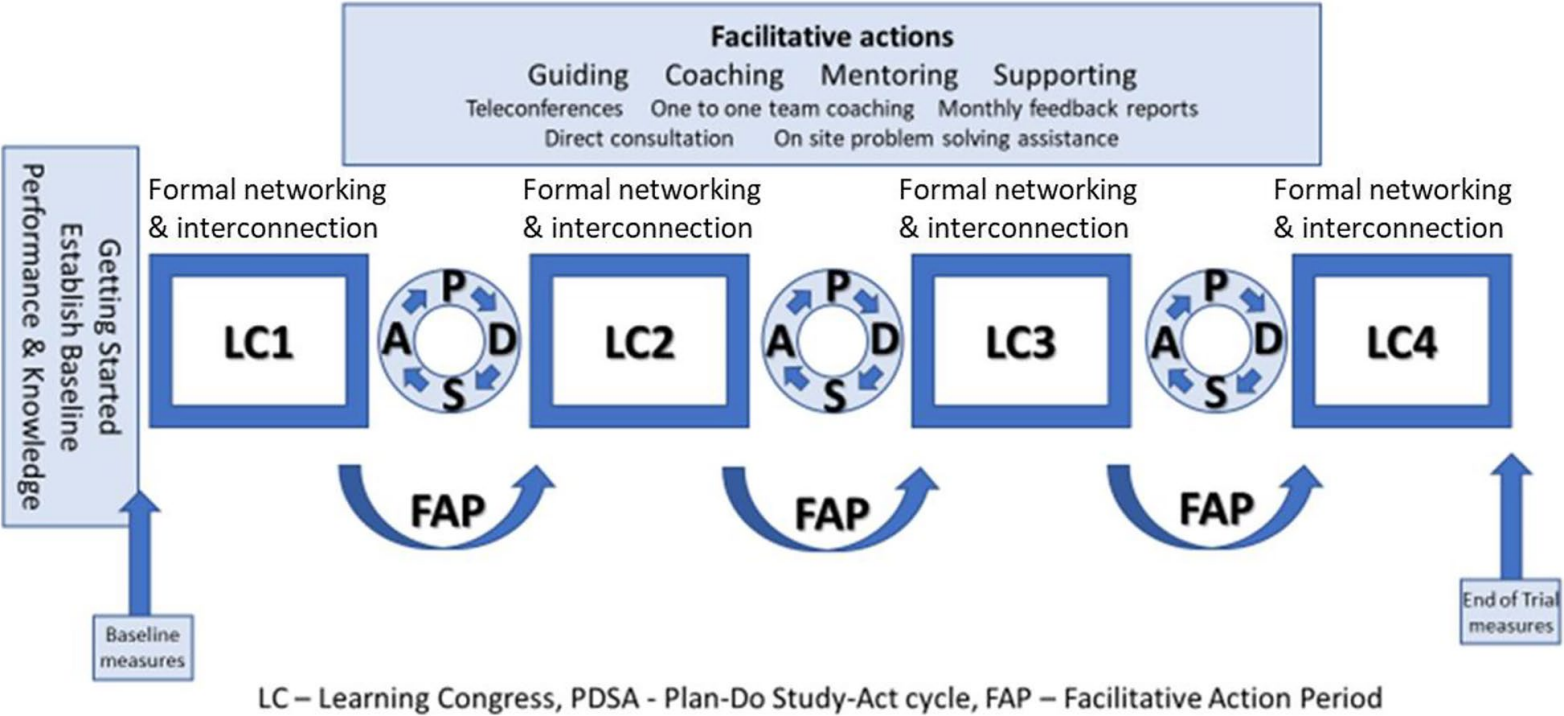
The SCOPE intervention

The SCOPE intervention is based on a modified Institute for Healthcare Improvement (IHI) Breakthrough Collaborative Series model designed around successful collaborative learning approaches for quality improvement [50, 51]. The intervention was developed, tested, and subsequently refined in an initial pilot study and a subsequent larger test for refinement for use with care aide led teams prior to this formal trial [48, 49]. The components were also informed by knowledge translation theory, with specific focus on the role that facilitation plays in implementation success [52, 53]. The intervention, including the facilitation activities in it described below, was delivered by regional Quality Advisors, supported by an overall study Coordinator that we called a Quality Coordinator. As shown in Fig. 1, the intervention comprised the following:

1. A “Getting Started Kit”: Teams and Sponsors (unit and facility managers) received baseline performance data on their selected clinical area, one of three—reducing pain, maintaining mobility, reducing responsive behaviours, selected by care aides as the most pressing problem within their LTC home in a Delphi process [54], information about how to modify the outcome (evidence from published literature supported by content written by experts in each area), and a SCOPE Change Package that introduced the Plan-Do-Study-Act (PDSA) cycle and QI concepts in the context of using best practice to improve resident care along with examples and models from the IHI collaborative model.
2. Four face-to-face “Learning Congresses” (LCs) brought together team members and sponsors from each region for one to one-and-a-half days every three months for networking and short plenary sessions and activities on the improvement model, measurement in PDSA cycles, team dynamics and function, engagement of colleagues in implementing ideas, and overcoming barriers to spread. The final congress was a celebration meeting, allowing teams to showcase their achievements and share experiences. Learning congresses were delivered primarily by the regional Quality Advisors and the Quality Coordinator.
3. Three “action periods” (improvement activity with ongoing coaching from Quality Advisors and the Quality Coordinator between LCs) when teams made small tests of change using Plan-Do-Study-Act (PDSA) cycles to improve care delivery in their selected clinical area and conducted small-scale measurement to gauge improvement. Teams used the IHI model for improvement comprising elements of successful process improvement: specific and measurable improvement aims, measures of improvement tracked over time, changes resulting in the desired improvement, and a series of testing “cycles” during which unit teams learned how to apply their ideas across their care unit. Teams implemented their change ideas with increasing numbers of residents on the unit during this time. PDSA cycles continued to adapt, adopt, or abandon change ideas throughout these action periods.
4. Participation in several facilitation activities to support QI during the action periods. Teams participated in a minimum of monthly teleconferences and a site visit facilitated by the regional Quality Advisors. Teams were supported in adoption and use of best practices, idea generation, measurement tools, analysis of results, and implementation challenges, with the degree of support tailored to the needs of each team.
5. A program of in-person learning sessions and online discussions on leadership, focused on “supporting and enabling change” for sponsors, delivered by the Quality Co-ordinator.

### Theoretical framing

The study was theoretically informed by the *Promoting Action on Research Implementation in Health Services* (PARiHS) framework [55]. This framework argues that successful implementation is a function of optimizing organizational context, facilitation (role or process), and evidence. The success of both QI collaboratives [56, 57] and implementation [58–63] depends on an optimized work environment (context) and sufficient facilitation, and here, implementation success was defined as the uptake and increased use of best practices by care aides [64–66]. In SCOPE, we defined facilitation capacity and its implied processes as *the capacity in an organization, specifically at the care home unit level, to engage in deliberate processes of interactive problem-solving in the context of a recognized need for improvement and supportive interpersonal and intra-organizational relationships*. The SCOPE intervention uses (1) *facilitation* (as a bundled set of activities) to support literacy in the language and tools of quality improvement, (2) *relationship building* to develop unit-based quality improvement teams that engage and are led by care aides, and (3) *interconnectedness* of these teams with other care providers on the unit, with senior management support and leadership. SCOPE also encouraged networking between teams from participating LTC sites as a form of collaborative learning.

Facilitation has been described both as a single intervention and as part of a multifaceted intervention [67]. A recent definition identified three elements of facilitation: project management, leadership and tailoring of facilitation efforts to the local context, and an emphasis on evaluation linking outcomes to actions [68]. Seers et al. [69] describe facilitation as existing on a continuum from *technical* to *enabling*. *Technical facilitation* focuses particularly on issues of implementation at the level of clinical teams. It encompasses designing systems and processes of care that enhance the transfer of evidence into day-to-day practice, and making use of techniques such as toolkits, both of which are features of the SCOPE intervention. A meta-analysis conducted in primary care found a relationship between intensity of facilitation and effect size in intervention studies [70]. However, we lack empirical evidence for the effectiveness of facilitation (as a role or process) on implementation, as well as knowledge about its operational mechanisms. The parallel process evaluation undertaken as part of SCOPE [71] attempted to generate real-world evidence and shed light on how change happened in SCOPE.

## Methods

SCOPE was a pragmatic controlled trial with each randomly selected LTC home identifying a care unit to participate in the intervention. Care aides and residents of these units (clustered within the units) formed the units of analysis. Control (usual care) units in non-intervention LTC homes were matched to intervention units post hoc (see below).

### Setting

This study was part of a larger research program examining modifiable contextual factors that influence implementation and improvement efforts in LTC homes in British Columbia, Alberta, and Manitoba, the Translating Research in Elder Care (TREC) program. TREC is a longitudinal research program comprised of many studies (including SCOPE). Its overall aim is to improve the quality of care and quality of life for LTC home residents and quality of work life for the staff who care for them [72]. TREC focuses on the level of the clinical microsystem (resident care units) where quality is created [73, 74]. The overall TREC cohort includes 94 urban homes and was created using a stratified (owner-operator model, size, region) random sample [72]. Homes participating in the SCOPE study were selected using a stratified random sample [72] of TREC homes in Alberta and British Columbia.

### Outcomes and measures

SCOPE was situated between two routinely occurring waves of TREC data collection and used variables from these sources for its quantitative outcome measurement. The primary outcome measure for this study aimed at improving use of best practices for resident care was between group change in *care aide-reported conceptual use of best practices* (*Conceptual Research Use, CRU*). *CRU is* defined as the cognitive, reflective use of research (best practices) where the knowledge may change one’s opinion or mindset about a specific practice area but not necessarily one’s direct actions. This scale asks about how often on a typical workday best practice knowledge helped with conceptual thinking about resident care, for example, by making sense of things related to resident care. It is an indirect application of research [75–78] measured using the five-item CRU scale. In reliability testing with care aides, Cronbach’s alpha for the 5-item CRU scale exceeded the accepted standard for scales intended to compare groups (alpha = 0.894) as well as acceptable response process, content, factorial, and construct validity [79–81].

### Secondary outcomes

Secondary outcomes included validated measures of care aide-reported outcomes on work engagement, job satisfaction, and burnout, and resident outcomes on clinical indicators for pain, mobility, and responsive behaviours collected as part of the Resident Assessment Instrument – Minimum Data Set (version 2.0) (RAI-MDS) [82]. All outcomes were collected at baseline and at the end of the SCOPE trial. Measures of the implementation fidelity, measured at the SCOPE team level in a concurrent process evaluation [71], were also collected. Only fidelity enactment data are incorporated into the main trial analysis reported here. Full details are shown in Table 1.

**Table 1:** Secondary outcome measures

**Care aide outcomes:** Collected at baseline and end of study, as part of the TREC care aide survey	**Burnout,** using the Maslach Burnout Inventory [83], for which adequate reliability (Estabrooks CA, Squires JE, Hayduk L, Morgan D, Cummings GG, Ginsburg L, et al: Does context matter? The influence of organizational context on best practice use by healthcare aides in residential long-term care settings, submitted) and validity are established (Estabrooks CA, Squires JE, Hayduk L, Morgan D, Cummings GG, Ginsburg L, et al: Does context matter? The influence of organizational context on best practice use by healthcare aides in residential long-term care settings, submitted) [84, 85]; **Job satisfaction** [86, 87] **Work engagement** [88]; **Psychological empowerment,** [89]; **Organizational citizenship behaviors** directed at the organization [90]	The work engagement, psychological empowerment, and organizational citizenship behaviour measures were adapted and validated for use with the care aide population [91]
**Resident outcomes:** All obtained from quarterly RAI-MDS 2.0 reports [92] included	(1) Physical functioning (Activities of Living—Hierarchy [ADL-H] scale score [93] (2) Responsive behaviours (Aggressive Behavior Scale [ABS] score of 2+) [94] (3) A pain score based on observable indicators of pain, which was developed and validated by TREC researchers to overcome the issue of under-detection of pain in residents with dementia [95, 96].	
**Implementation fidelity:** measured at the level of the SCOPE team	**Fidelity enactment** (intervention participants actual performance of intervention skills/implementation of the core intervention components in the intended situation) was assessed on a four-point scale by a panel of investigators at the final celebration learning congress. The panel rated each team’s actual implementation of SCOPE activities: defining aims, generating change ideas, using PDSA cycles and measurement to test changes, modifying unsuccessful changes, spreading successful changes to residents and staff across the unit. The single-item global fidelity enactment measure was developed in a previous study [97] and adapted for use in the SCOPE pilot study [49, 98]	

### Sample size and power calculation

The primary outcome measure was change in Conceptual Research Use (CRU), from baseline to post intervention, compared between intervention and control (usual care) units. Initial modeling was based on unit aggregate expected change in the primary outcome, dictating a sample size of 34 units to be matched to usual care units, but was replaced by a care aide level analytical model, deviating from the original published trial protocol (NCT03426072). Thus, for an effect size of *d*=0.22 (based on a mean difference of 0.11 in the CRU score between the intervention and control group at follow-up and a standard deviation of 0.5 in both groups, informed by our pilot data [40]), the required sample size was 652 CA surveys, *n*=326 in each study group (based on a two-tailed test for independent study groups, at 80% power, with an alpha of 0.05). Considering possible clustering effects, we multiplied this required sample size by a variance inflation factor (VIF=1+ [cluster size – 1] *intra-cluster correlation). Based on previous TREC data, we assumed a cluster size of 15 care aide surveys per unit and an intracluster coefficient of 0.01. Therefore, our required sample size was 652*1.14=744 care aides (*n*=372 per study group) or 50 care units (25 in each study group, each providing an average number of 15 care aide surveys).

### Sampling

To be eligible to participate, LTC homes had to (a) be a part of the TREC cohort in Alberta and British Columbia; (b) have units comprising general nursing care for older adults, rather than those co-managed with acute care; (c) have the majority of residents over the age of 65; (d) have more than 35 beds in total; (e) be geographically located within 100km of either Edmonton: Edmonton Health Zone (EH) or Calgary: Calgary Health Zone (CH) in Alberta (AB), or Kelowna: Interior Health (IH), or New Westminster: Fraser Health (FH) in British Columbia (BC); (f) use the Resident Assessment Instrument-Minimum Data Set 2-0 (RAI-MDS) to gather resident level care indicators; and (g) have 8 or more care aide responses to the baseline trial data collection survey.

Eligible LTC homes were stratified by region (EH, CH, IH, FH), owner operator model (for profit, not for profit), and size (small: <80 beds, medium: eight-120 beds, large: >120 beds), and randomly selected for participation. Based upon feedback from decision-makers and LTC home administrators, it was decided that randomization to intervention or control at the outset would not be feasible because of the likelihood of bias favouring refusal to participate as an inactive “control” site. Thus, *random selection* was undertaken only for intervention sites, with replacement for refusals. Once the number of LTC homes within the same stratum was exhausted, a replacement home was randomly selected from the remaining homes in that region.

Because of the limited number of eligible LTC homes in the cohort, homes which declined to participate were returned to the main TREC cohort to act as usual care (control) comparators. After removing ineligible units (those who did not participate in both the baseline and follow up data collections and those with fewer than eight care aide responses to the TREC care aide survey) to ensure stability of measures at either the baseline or follow up data collections, we randomly matched a control unit to each intervention unit, based on the unit type: general long-term care versus dementia care unit, number of beds on unit, facility size category (small: <80 beds, medium: eight-120 beds, large: >120 beds), ownership model (for-profit, not-for-profit), and region.

Directors of care in charge of each selected home were invited to participate, provided with information about the study, and included if they consented to participate. Homes were provided with $3000 as partial compensation for the time and resources required to participate. Following discussion of trial requirements, Directors of Care were given the task of identifying one care unit within their home to participate in the intervention and to identify staff as members of their SCOPE team.

### Participants

SCOPE teams comprised four to seven members, at least two of which were care aides. Each team was either led by a care aide or co-led by two care aides. Other team members consisted of unit-based care aides and/or professional staff (e.g*.*, registered nurse, physiotherapist, occupational therapist, recreation therapist). A team sponsor (usually a unit-level clinical nurse manager) was responsible for supporting day-to-day project activities. A senior sponsor, normally at the facility Director of Care or the care manager level in large units, agreed to actively support each team, removing barriers to change, and supporting time spent on quality improvement.

### Analysis

SAS® 9.4 (SAS Institute Inc., Cary, NC, USA) was used for all statistical analyses. Using descriptive statistics, baseline characteristics of LTC homes, care units, care aides, and residents were compared between study arms. To assess intervention effectiveness, mixed effects regression models were used [99, 100]. All models were adjusted for sampling strata, baseline differences of the outcome variables, care aide characteristics (sex, age, English as first language [yes/no]), and care unit staffing (total care hours per resident day and percentage of total hours per resident day provided by care aides). A unit-level random intercept was added to account for dependencies of responses provided by care aides on the same care unit. Similar models were used to assess the impact of the intervention on resident outcomes but adjusted for resident characteristics (age, sex, case mix index). Finally, to assess whether improvements in outcome scores were higher in intervention facilities with higher (above median) enactment scores, mixed effects regression models were used, adjusted for the same variables as above, and included an interaction term between the dichotomous enactment variables (high/low) and data collection (baseline/follow-up).

## Results

A total of 31 LTC homes were randomly selected to form the intervention group. The intervention took place between May 2018 and May 2019. While facilities were asked to select only one care unit to participate in the study, one home included three of their care units and another included two units, so the intervention group baseline sample was 34 care units in 31 LTC homes. Two facilities withdrew their participation during the study, citing external pressures. The final sample included 32 care units in 29 LTC homes (total of 1221 beds) in the intervention group and 32 care units in 30 LTC homes (total of 1258 beds) in the control group. From these care units, 1719 surveys from care aides (866 at baseline and 853 at follow-up) were collected. Seven SCOPE homes (two AB, five BC) engaged with quality improvement projects on pain, seven (five AB, two BC) on maintaining mobility, and 17 (seven AB, ten BC) addressed responsive behaviours. Descriptions of participating LTC homes are in Table 2. Twenty-seven of the 32 teams (84%) participated in all four learning congresses [details of team participation and implementation processes are provided in the companion paper [71].

**Table 2:** Facility, care unit, and care aide characteristics at baseline by study arm

	Intervention	Control
**Facility sample**		
**Number of facilities**	29	30
**Region**		
Calgary	6 (20.7%)	7 (22.3%)
Edmonton	6 (20.7%)	7 (22.3%)
Fraser health	11 (37.9%)	11 (36.7%)
Interior health	6 (20.7%)	5 (16.7%)
**Size**		
Small	9 (31.0%)	4 (13.3%)
Medium	7 (24.1%)	12 (40.0%)
Large	13 (44.8%)	14 (46.7%)
**Ownership**		
Public	5 (17.2%)	6 (20.0%)
Voluntary	10 (43.5%)	10 (33.3%)
Private	14 (48.3%)	14 (46.7%)
**Unit sample**		
**Number of units**	32	32
**Unit type**		
General LTC	21 (65.6%)	19 (59.4%)
Secure dementia/mental health	7 (21.9%)	8 (25.0%)
Other	4 (12.5%)	5 (15.6%)
**Unit staffing**, M ± SD hours per resident day		
Care aides	1.8 ± 0.5	1.8 ± 0.7
Licensed practical nurses	0.4 ± 0.3	0.6 ± 0.4
Registered nurses	0.3 ± 0.2	0.3 ± 0.2
Total staffing	2.5 ± 0.6	2.7 ± 0.9
**Care aide sample**		
**Number of care aides**	441	440
**Females**	411 (93.2%)	393 (89.3%)
**Age category**		
< 25 years	19 (4.3%)	25 (5.7%)
25–34 years	73 (16.6%)	70 (10.5%)
35–44 years	109 (24.7%)	118 (26.8%)
45–54 years	127 (28.8%)	138 (31.4%)
> 54 years	113 (25.6%)	89 (2.2%)
**English as second language**	283 (64.2%)	280 (63.6%)
**Short staffed at least weekly**	162 (36.7%)	180 (40.9%)
**Years worked on unit**, M ± SD	6.0 ± 6.2	5.6 ± 5.8
**Years worked as care aide**, M ± SD	12.1 ± 9.5	10.9 ± 8.7
**Resident sample**		
**Number of residents**	1438	1397
**Age**, M ± SD		
**Females**	903 (62.8%)	928 (66.4%)
**Married**	379 (26.4%)	366 (26.2%)
**Moderate to severe cognitive impairment**	865 (60.2%)	960 (68.7%)
**Moderate to severe physical impairment**	1125 (78.2%)	1147 (82.1%)

*Notes*: *M* mean, *SD* standard deviation

There was no statistically significant difference in any of the outcome measures between intervention units and control units at baseline: all *p*>0.05 (Table 3).

**Table 3:** Study outcomes (unadjusted M ± SD) by study arm at baseline and follow-up

	Baseline		Follow-up	
	Intervention	Control	Intervention	Control
**Number of care aides**	441	440	412	426
**Number of residents**	1438	1397	1390	1281
**Primary outcome**				
Conceptual research use	4.0 ± 0.8	4.1 ± 0.7	4.1 ± 0.8	4.1 ± 0.7
**Secondary care aide outcomes**				
Job satisfaction	4.3 ± 0.6	4.2 ± 0.6	4.2 ± 0.6	4.2 ± 0.7
Work engagement vigor	5.4 ± 0.9	5.4 ± 0.9	5.3 ± 0.9	5.3 (1.0)
Work engagement dedication	5.6 ± 0.7	5.6 ± 0.7	5.6 ± 0.7	5.6 ± 0.7
Work engagement absorption	5.8 ± 0.4	5.8 ± 0.4	5.8 ± 0.5	5.8 ± 0.5
Organizational citizenship behaviour	3.8 ± 0.7	3.7 ± 0.6	3.7 ± 0.6	3.8 ± 0.6
Often suggest to coworkers ways to improve their work	3.9 ± 0.8	3.8 ± 0.8	3.8 ± 0.8	3.9 ± 0.8
**Secondary resident outcomes**				
ADL-H score	3.5 ± 1.5	3.7 ± 1.4	3.7 ± 1.4	3.9 ± 1.4
ABS score	1.0 ± 1.9	1.3 ± 2.3	1.0 ± 1.8	1.3 ± 2.2
Pain score	1.0 ± 1.5	1.2 ± 1.6	0.9 ± 1.4	1.2 ± 1.6

*Notes*: *ABS* Aggressive Behaviour Scale, *ADL-H* Activities of Daily Living Hierarchy scale

### Primary outcome

In the adjusted model (Table 4), there was no statistically significant difference between intervention and control units for CRU at follow-up. However, the CRU score in the intervention group increased by 0.09 points at follow-up (*p*=0.07) but remained constant in the control group.

**Table 4:** Adjusted mean differences [95% confidence interval] of study outcomes based on mixed effects models

	Intervention—control at follow-up	Post—pre, intervention	Post—pre, control
**Primary outcome**			
Conceptual research use	0.004 [−0.110; 0.119]	0.088 [−0.008; 0.183]	−0.003 [−0.098; 0.091]
**Secondary care aide outcomes**			
Job satisfaction	0.004 [−0.096; 0.105]	0.006 [−0.076; 0.089]	0.036 [−0.046; 0.118]
Work engagement vigor	0.031 [−0.118; 0.181]	−0.040 [−0.163; 0.084]	−0.069 [−0.192; 0.053]
Work engagement dedication	−0.015 [−0.123; 0.093]	−0.002 [−0.099; 0.094]	−0.017 [−0.112; 0.078]
Work engagement absorption	−0.027 [−0.094; 0.041]	−0.041 [−0.102; 0.020]	−0.028 [−0.088; 0.033]
Organizational citizenship behaviour	−0.098 [−0.202; 0.007]	−0.033 [−0.116; 0.050]	0.079 [−0.003; 0.161]
Often suggest to coworkers ways to improve their work	−0.125 [−0.256; 0.006]	−**0.114 [**−**0.223;** −**0.004]**	0.098 [−0.011; 0.206]
**Secondary resident outcomes**			
ADL-H score	−0.118 [−0.305; 0.069]	**0.182 [0.093; 0.271]**	0.066 [−0.026; 0.158]
ABS score	−0.253 [−0.677; 0.171]	−0.034 [−0.173; 0.105]	−0.062 [−0.207; 0.083]
Pain score	−**0.373 [**−**0.698;** −**0.048]**	−**0.137 [**−**0.233;** −**0.031]**	0.041 [−0.064; 0.146]

### Secondary staff outcomes

**Fig. 2:**
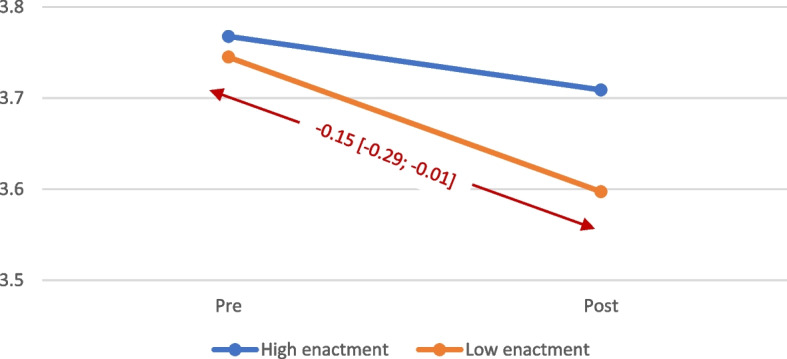
Change in scores measuring how often care aides suggested ways to improve performance to their colleagues by level of enactment based on mixed effects models

There was no statistically significant difference in any of the secondary staff outcomes, based on adjusted mean scores at follow-up between the intervention and control group (Table 4). A post hoc analysis of item two of the Organizational Citizenship Behaviours (OCB) scale: “I often suggest to my co-workers new ways about how to improve the work on the unit” revealed a statistically significant decrease in score (worsened) at follow-up, compared to the baseline, in the intervention group (*p*=0.04). Analysis of the intervention units including an interaction term between high/low intervention fidelity enactment and time of data collection showed that, over the duration of the intervention, the score on this OCB item decreased statistically significantly only on intervention units with low levels of enactment, while on care units with high levels of enactment the decrease was not statistically significant (Fig. 2). Enactment was not associated with other study outcome.

### Resident care outcomes

No intervention effects were found for those teams working on responsive behaviors. However, the adjusted level of resident dependency significantly decreased for residents whose teams addressed mobility (*p*<0.0001) at follow-up, compared to the baseline, in the intervention group. While adjusted follow-up scores in the intervention group for those teams working on resident pain were higher than at baseline (*p*=0.01), adjusted pain scores at follow-up were lower (indicating less pain) in the intervention group compared to the control group (*p*=0.02).

## Discussion

SCOPE was a multicomponent intervention designed to facilitate the use of best practices in care for older LTC home residents. SCOPE incorporated elements of design to support sustainability and address the need for programs of research on implementation and improvement in healthcare [101, 102]. The facilitated SCOPE intervention aimed to empower and enable care aides to lead QI initiatives using PDSA cycles as the vehicle with which to test small changes in care processes, increasingly considering and therefore using best practices. Our expectation was that this would also lead to improvements in our secondary outcomes—care aide job satisfaction, work engagement, and ultimately to improvements in the quality of resident care delivered [103–106].

### Primary outcome

SCOPE was initially planned as a randomized clinical trial, attempting to take the complex nature of the system in which it was implemented into account. Using quantitative measures, SCOPE’s primary aim, improvement in the conceptual use of best practices, was not demonstrated. The intention was that teams would implement what they had learned by taking part in SCOPE, developing, and testing small changes in care practices, based upon best practices in care, which would then be spread across the unit and embedded in usual care. The outcome, Conceptual Research Use (use of best practices), was thought to best capture the essence of the change in thinking resulting from the team quality improvement collaborative and theoretically links beliefs regarding research use being predictive of actual best practice use [107]. SCOPE resulted in a smaller change in its primary outcome than initially expected, with an effect size smaller than that obtained in our pilot project [40], resulting in a study statistically underpowered to detect a difference.

### Secondary outcomes—staff

There were no statistically significant group differences in staff-related secondary outcomes. These findings should inform sample size calculations of future studies of this nature for example, if based on the size of effects we report using similar outcomes measures.

A post hoc analysis revealed a statistically significant improvement in care aide perspectives on new ways of working on the unit (one element of OCB) and a positive relationship between SCOPE fidelity enactment and these behaviors. This last finding reinforces the importance of recent Medical Research Council guidance [108] and a systematic review [109] which suggests that conducting process evaluations and considering implementation are important in complex trials such as SCOPE and may well be mandatory to describe resulting change.

In addition to the, retrospectively, smaller than needed sample size, our concurrent process evaluation [71, 108] affords valuable and rarely available insights into other possible explanations for finding no measurable difference between intervention and control units for most of our outcomes [110]. Firstly, the primary outcome, conceptual research use, relied on teams using best practices (with facilitation) to inform their small tests of change. However, many tested changes on the ground were more pragmatic (such as removing a door in the dining room to address disruption) and may have only been indirectly related to best practices.

Secondly, SCOPE teams consisted of four—six members, actively working on their PDSA cycles, and attempting to improve quality of care; the team members had to spread their improvement efforts across the entire unit, ensuring that “new ways of doing” were adopted by care providers who were not part of the SCOPE team. It is possible that adoption was not spread sufficiently *across* staff on the unit who provided data on the primary and secondary outcomes. Outcome data were also collected from care aides on the unit who may not have been SCOPE team care aide members. Our process evaluation data [71] suggested that SCOPE teams often had difficulty spreading SCOPE to all staff on a SCOPE unit. Given increasing recognition of the minimal impact of QI, insights from concurrent process studies are important [111].

### Secondary outcomes—residents

Examination of two of the three resident care indicators from the RAI-MDS showed no difference in change between groups over the period of observation. The finding in pain assessment is however, encouraging, suggesting that teams were successful in improving the quality of care for residents in pain, but this is a single finding in need of replication. There may be several reasons for the inability to detect a change in resident outcome measures. Firstly, the SCOPE intervention was implemented over a year and most teams took time to function effectively, to design their aim statements and conduct tests of small changes to improve quality [71]. Effects relating to these changes conceivably may take far longer to be detectable in RAI-MDS data [102]. Secondly, teams addressing, for example, responsive behaviours often targeted specific times of the day when they found these more problematic (e.g., mealtimes). Such endeavours were likely insufficient to result in a degree of decline in behaviours across the unit to move the RAI-MDS 2.0 indicator. Thirdly, in many units, teams felt only a proportion of residents were appropriate with whom to work, potentially leaving most residents with “usual care,” further diluting the effect of the intervention. Finally, given the nature of the LTC home resident population (near end of life with a progressive set of chronic conditions, such as dementia), avoidance of a decline in mobility, rather than an improvement, is likely a more meaningful indication of success.

For all outcomes and teams, SCOPE was dependent upon leadership sufficiently able to support change. Leaders were called upon to remove obstacles, provide sufficient time for QI initiatives and to allow the care aide leaders to lead the team. This was variably achieved, with some leaders adopting and maintaining a more authoritarian approach to SCOPE implementation, providing less than sufficient resources and time for teams to meet, potentially adding to work life pressures, rather than relieving them [71]. “Top-down” approaches have markedly different effects to “bottom-up” methods in improvement collaboratives [112]. Other findings from the concurrent process evaluation provide important insights into SCOPE implementation that can help with interpretation of the main trial data presented here and deepen our understanding of how teams implement complex interventions in LTC home settings [71, 108]. The process evaluation results also suggest the need to consider more broadly what constitutes “success” in complex interventions.

### Limitations

We note above limitations related to the primary and secondary outcomes. There are additional important limitations. While initially intended as a randomized controlled trial with propensity matching to TREC units not participating in SCOPE, two problems were encountered that were eventually insurmountable, resulting in the quasi-experimental design reported here. Firstly, the number of LTC homes in the sampling frame was exhausted before the intended sample size was reached. Secondly, to maintain the size of our control cohort in a potential 1:1 match, we had to return homes that had declined to participate to the general pool, potentially introducing a bias to the comparator group. Despite the close working relationships established with many of the homes, and our previous experience with this intervention, recruitment was more challenging than originally anticipated [113]. Because of these factors and the fact that, to avoid refusal bias, as advised by our nursing home leaders, we were unable to randomize a priori, selection of control units was less than optimal. However, variables did not vary across all TREC units at baseline, and matching was carefully performed at the analytical stage. The trial was also methodologically limited in that although intervention homes were randomized, and the care units where SCOPE was implemented (the units of analysis) were not. The selection of care units was at the discretion of Directors of Care, who best knew their units and staff, potentially introducing selection bias and perhaps bias to trial success. The variability in achieved results across the intervention cohort suggests that local knowledge in unit selection did not produce such bias.

## Conclusion

In conventional terms, SCOPE was a negative study further contributing to the file drawer problem [114] and highlights the problem with measures drawn from current LTC databases to capture change in this population. However, our concurrent process evaluation and its attention to important concepts such as fidelity and implementation, as well as its attention to how the trial was experienced by care staff, suggests a markedly different picture in which staff interpreted their participation positively and evaluated its success quite differently. This causes us to question how we define and measure success in such trials. The process evaluation enables us to draw conclusions about the true value of SCOPE that would have previously gone unrecognized. Consequently, we can consider critical questions that are generally poorly examined, such as how and why the SCOPE intervention worked, or failed to work.

## Data Availability

The data used for this article are housed in the secure and confidential Health Research Data Repository (HRDR) in the Faculty of Nursing at the University of Alberta (https://www.ualberta.ca/nursing/research/supports-and-services/hrdr), in accordance with the health privacy legislation of participating TREC jurisdictions. These health privacy legislations and the ethics approvals covering TREC data do not allow public sharing or removal of completely disaggregated data from the HRDR, even if de-identified. The data were provided under specific data sharing agreements only for approved use by TREC within the HRDR. Where necessary, access to the HRDR to review the original source data may be granted to those who meet pre-specified criteria for confidential access, available at request from the TREC data unit manager (https://trecresearch.ca/about/people), with the consent of the original data providers and the required privacy and ethical review bodies. Statistical and anonymous aggregate data, the full dataset creation plan, and underlying analytic code associated with this paper are available from the authors upon request, understanding that the programs may rely on coding templates or macros that are unique to TREC.

## Abbreviations

AB: Alberta
ABS: Aggressive Behaviour Scale
ADL-H: Activities of Daily Living Hierarchy
BC: British Columbia
CH: Calgary Health Zone
CRU: Conceptual research use
EH: Edmonton Health Zone
FH: Fraser health
IH: Interior health
IHI: Institute for Healthcare Improvement
LTC: Long-term care
RAI-MDS: Resident Assessment Instrument - Minimum Data Set
OCB: Organizational citizenship behaviors
PARiHS: Promoting Action on Research Implementation in Health Services
PDSA: Plan-do-Study Act
QA: Quality advisor
QI: Quality improvement
SCOPE: Safer Care for Older Persons in residential Care Environments
TREC: Translating Research in Elder Care
